# Progress of Surgery

**Published:** 1904-02-06

**Authors:** 


### Progress of Surgery.

*Concluded from page 316.*

**Traumatic Uræmia, cured by Cystotomy.**—B. C. Stevens[5] records the case of a lad, aged 16, who received a severe contusion of the abdomen. He was unconscious and in a state of shock on admission to hospital. A catheter was passed into the bladder and bloody urine drawn off. On the third day he regained consciousness at intervals but soon lapsed into coma again, and the condition then appeared to be one of uræmic poisoning. There was at this time a swelling in the hypogastrium reaching up to the umbilicus. A few ounces only of bloody urine could be then drawn off with a catheter. Suprapubic cystotomy was performed and the bladder found to be two-thirds full of organised clot, which was very adherent and could only be cleared out with difficulty. A fortnight later a cast of a large part of the bladder was passed through the abdominal wound. The patient was discharged well at the end of a month. The case is somewhat rare and shows that when the bladder fills with blood, which cannot be voided easily, this blood can produce uræmia by back pressure on the kidneys. The cast would appear to have been a slough produced by the initial contusion.

**Operation for Movable Horseshoe Kidney.**—A. Sturmdorf[6] reports a case of this kind. Horseshoe kidney, according to H. Morris occurred once in 1,600 cases. The patient, a woman, complained of a dragging sensation in the lower dorsal spine, with constant pain radiating downwards and forwards. The pain would subside on lying down, but the patient was never entirely free from it. She had had the symptoms for two years. Several times she had experienced sudden exacerbation of the pain during which there was anuria followed later by polyuria. A solid mass was felt occupying the right side of the abdomen, and seeming to fill the right loin, it could be moved freely up and down, but had not the shape of the kidney, and could not be made to slip into position on bimanual pressure in the manner characteristic of floating kidney. On making a lumbar incision, the upper pole of the kidney was easily liberated, but the lower pole could not be isolated because of its projection inward beyond the reach of the finger. The peritoneum was opened and a perfectly characteristic mobile horseshoe kidney thus exposed. The kidney was fixed according to Senn's method with gauze packing; this yielded firm fixation and the patient was completely relieved. The case could not be properly classed as a floating horseshoe kidney because the right segment moved so independently of the left. Probably it was a congenitally displaced horseshoe kidney the right part of which had become in some way detached.

**Impacted Calculus and Some Other Causes of Obstruction in the Ureters.**—P. J. Freyer[7] has published a clinical lecture on the above subject. He stated that it was only when the stone was impacted in the lower end of the ureter that a definite diagnosis could be ventured upon. The three places where a stone was most likely to get impacted were: (1) at a point 2 or 3 inches from the beginning of the ureter; (2) at the brim of the pelvis; and (3) at the vesical end of the canal. The author knew of no symptoms by which with any certainty one could distinguish between a stone lying in the pelvis of the kidney and one impacted in the ureter in any position above an inch or two from its vesical orifice. The symptoms except in the last case were practically the same as those of a stone in the renal pelvis. A stone in the ureter might give rise to pain or tenderness at the seat of impaction, and *x*-rays might assist in forming a diagnosis, but a definite diagnosis being so difficult it was the best practice in such cases to explore the kidney in the first instance, and if the stone were not found, then to examine the ureter with a bougie or probe. The impaction of the stone in the vesical end of the ureter should be suspected if after the subsidence of attacks of renal colic bladder symptoms persisted and a loose stone could not be found in the bladder to account for the symptoms. In such cases the stone should be felt for per rectum or per vaginam. If the stone projected from the ureteral orifice into the bladder it might be felt with the sound, or in the female it might be felt with the finger passed into the bladder, or in either sex the cystoscope might reveal its presence. Freyer then gives an account of seven cases of impacted stone in the ureter upon which he had operated. In two of these the stones were within a few inches of the kidney, and were removed by incision of the ureter over the stone. Of stones in the lower end of the ureter one was removed by vaginal uretero-lithotomy, one by retroperitoneal lithotomy, another by suprapubic cystotomy, and a fourth was dragged out of the ureteral orifice with a lithotrite after being seen clearly with the cystoscope. In one case a stone was found to lie in a tumour projecting from the posterior aspect of the bladder and attached to it by a broad and short pedicle. The cyst was apparently a bulging of the ureteral wall into the bladder caused by the impaction of the stone. The symptoms had been those of severe vesical irritation and cystitis. Discussing intra-peritoneal removal of ureteral calculus the author alluded to the great danger there was of the urine leaking into the peritoneum, no matter how carefully the wound in the ureter had been sutured. The method was one he would not adopt. He preferred the extra-peritoneal removal of a stone through an incision in the groin to that by the vaginal route. The latter was liable to be followed by a permanent urinary fistula, and had other drawbacks. After removing a stone it was not advisable to close the ureteral wound with sutures. The difficulty of diagnosing stone in the ureter was increased by the fact that other causes of obstruction of the canal gave rise to similar symptoms. Such conditions were: valvular obstructions, compression of the ureter by fibrous bands or an abnormal renal vessel, a twist of the ureter due to movable kidney, and stricture, stenosis, and kinking of the ureter. A stricture should be treated by longitudinal incision upon a bougie inserted in the ureter and suture of the wound in the transverse direction. A kink or twist should be relieved and fixation of the kidney high up performed. A valvular formation should be incised through an incision in the renal pelvis or a plastic operation performed.

[5] Lancet. Aug. 29, 1903, p. 606. [6] Med. Rec., Aug. 8, 1903, p. 231. [7] Lancet, Aug. 29, 1903, p. 585.

